# The Rare Coincidence: Nonrecurrent Laryngeal Nerve Pointed by a Zuckerkandl's Tubercle

**DOI:** 10.1155/2012/143049

**Published:** 2012-03-26

**Authors:** Emin Gurleyik, Sami Dogan, Omer Gunal, Mevlut Pehlivan

**Affiliations:** Department of Surgery, Medical Faculty, Duzce University, 81000 Duzce, Turkey

## Abstract

The safety of thyroid operations mainly depends on complete anatomical knowledge. Anatomical and embryological variations of the inferior laryngeal nerve (ILN), of the thyroid gland itself and unusual relations between ILN and the gland threaten operation security are discussed. The patient with toxic multinodular goiter is treated with total thyroidectomy. During dissection of the right lobe, the right ILN which has nonrecurrent course arising directly from cervical vagus nerve is identified and fully isolated until its laryngeal entry. At the operation, we observe bilateral Zuckerkandl's tubercles (ZTs) as posterior extension of both lateral lobes. The left ILN has usual recurrent course in the trachea-esophageal groove. The right ZT is placed between upper and middle third of the lobe points the nonrecurrent ILN. The coincidence of non-recurrent ILN pointed by a ZT is rare anatomical and embryological feature of this case. Based on anatomical and embryological variations, we suggest identification and full exposure of ILN before attempting excision of adjacent structures, like the ZT which has surgical importance for completeness of thyroidectomy.

## 1. Introduction

The thyroid has a lot of embryologic and anatomic variations affecting the safety of total thyroidectomy which is the procedure of choice for treatment of surgical disease of the thyroid. Therefore, a thyroid surgeon must have complete knowledge of all anatomic variations of the gland and of adjacent structures. As the inferior (or recurrent) laryngeal nerve (ILN or RLN) is the most important structure in terms of complication, anatomic variations of the nerve may threat the safety of the thyroid surgery. Nonrecurrent ILN is a rare but important example of anatomic variations [1–3]. 

 Emil Zuckerkandl (1849–1910), an Austrian anatomist, has described posterior extension of lateral lobes of the thyroid gland [4, 5]. The tubercle should be included in the Nomina Anatomica as the “processus posterior glandulae thyroideae” described by Zuckerkandl [6]. Zuckerkandl's tubercle (ZT) has a close relationship with the RLN at its distal part. ZT is a common structure which is found during operations in many patients with goiter [7–10]. As the nonrecurrence of inferior laryngeal nerve is a rare variation, the coexistence of ZT and non-RLN is rarely observed during the operation. In PubMed, the authors could not find any previously reported coexistence of non-RLN and the ZT in the same patient.

 In this manuscript, we present the coincidence of ZT and non-RLN in a patient with toxic multinodular goiter treated with total thyroidectomy.

## 2. Case Report

 A 66-years-old female patient with goiter had a history of thyroid gland enlargement for a long time. The patient presented our department with symptoms of hyperthyroidism. Physical examination, blood chemistry for hormones profile, ultrasound imaging, and thyroid scintigraphy established the diagnosis of toxic multinodular goiter. Thyroid hyper-function was normalized with antithyroid medication before operation.

**Figure 1:**
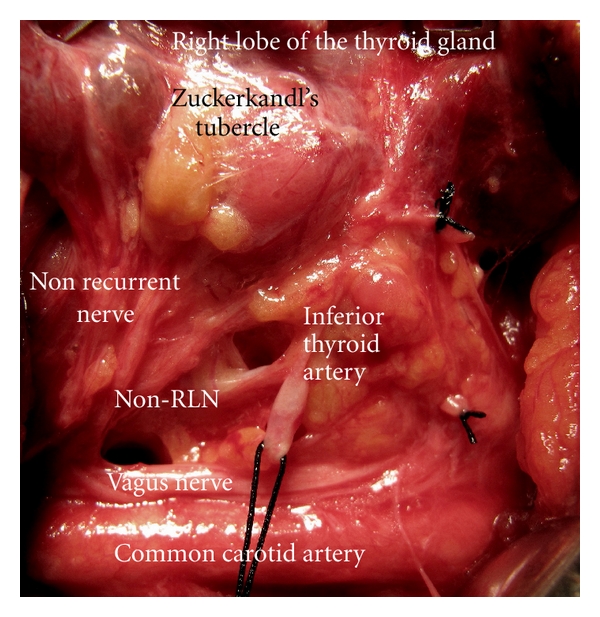
The right Zuckerkandl's tubercle as a posterior extension of the right lateral lobe, points the nonrecurrent inferior laryngeal nerve (non-RLN). It arises from the vagus nerve and courses directly to laryngeal entry.

**Figure 2:**
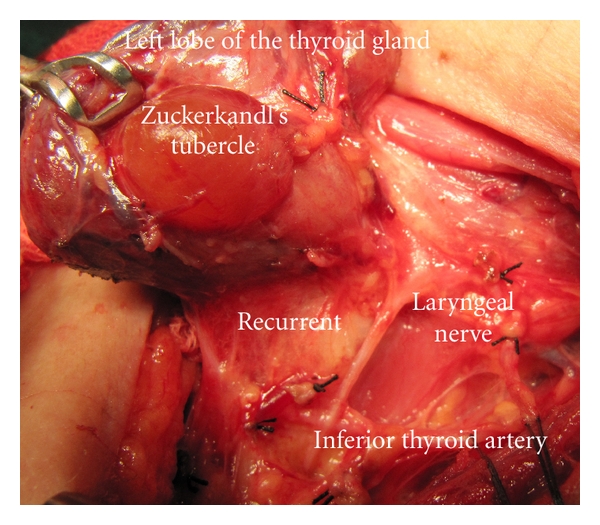
Left side of the same patient; the left Zuckerkandl's tubercle is present as posterior extension of the left lateral lobe. The recurrent laryngeal nerve is at usual position.

 The right lobe is mobilized medially after ligation and cutting the middle thyroid vein and branches of superior thyroid artery closely to the gland. Between superior and middle third of the lobe, we observe posterior extension of the tissue; a grade 3 (larger than 10 mm) ZT is present in the right lateral lobe. The inferior thyroid artery is identified, isolated, and a loop of silk suture is placed around the artery for traction after freeing and medially mobilizing the thyroid gland. We use this artery as an anatomical landmark to identify the ILN. Using standard lateral approach, the right ILN is explored at anticipated crossing point of the nerve and the artery. The right RLN is not found at its usual course, and the dissection is advanced upward direction. A nonrecurrent laryngeal nerve is identified and exposed near the Berry ligament. The nerve is fully isolated from its originating point on the vagus nerve until its laryngeal entry. The nonrecurrent nerve is showing a parallel course with the inferior thyroid artery. It is placed at 3 cm cephalic position to the artery. The ZT situated at posterior side between upper and middle third of the right lobe is pointing the non-RLN (Figure 1). This is a coincidence of a rare variation of ILN and posterior extension of lateral lobe of the thyroid gland. Exploration of the left lateral lobe also shows the presence of a left ZT. Our patient has posterior extension in both lateral lobes: bilateral Zuckerkandl's tubercles. The left RLN which has usual recurrent course is also fully isolated before resection of the gland (Figure 2).

## 3. Discussion

 The safety of the thyroid operation mainly depends on the surgical anatomy of the ILN. Complete knowledge of RLN anatomy including all its variations is mandatory for thyroid surgeons. Nonrecurrent ILN is a rare but important variation affecting safety of thyroid operations. The prevalence of nonrecurrent nerve has been reported less than 1% [2, 3]. Full exposure of RLN is required for avoiding nerve injury. The site of greatest risk during thyroidectomy to the nerve is in the distal 2 cm of its course above the trunk of the inferior thyroid artery where the nerve is more superficial and vulnerable to injury [11].

 Emile Zuckerkandl has been reported in 1902 “processus posterior glandulae thyroidea” [4, 5]. Many authors have recently reported the incidence of ZT as more than 50% of their patients [7, 8, 12]. Gauger et al. [8] have reported bilateral ZT in 15% of their patients. Therefore, like our patient, bilateral ZTs is less common finding. Pelizzo et al. [13] have classified the ZT into 3 grades according to size; as the diameter of the ZT, <5 mm (grade 1), <10 mm (grade 2), and larger than 10 mm (grade 3). The tubercle is generally enlarged in case of (goiter formation) thyroid hypertrophy. The completeness of thyroidectomy requires total removal of enlarged tubercle. Our surgical dissection and medial mobilization of the right lobe has displayed posterior extension of thyroidal tissue between upper and middle third of the lobe. Yalçin et al. [14] have reported the location of the tubercle of which 88% was located in the middle and only 4% in the upper third of the lateral lobe. In our patient, the location of ZT is found slightly upper position than the majority of reported cases.

 Beside completeness of thyroidectomy, importance of ZT mainly arises from its relation (neighboring) with ILN. An understanding of the relationship between the ZT and RLN is essential for safety of thyroid operations [8, 10, 12]. The resection of enlarged tubercle at posterior site of the thyroid requires delicate and careful dissection adjacent to the nerve. Some anatomical landmarks help surgeons identifying RLN. The inferior thyroid artery is an important landmark in all our operations. In our technique, we use inferior thyroid artery as a constant landmark for identification of the nerve by lateral approach after traction of the artery with a loop of silk. The ZT when present contributes to the identification of RLN. Many authors have previously stated that the ZT is a reliable and constant anatomical landmark as an arrow pointing the RLN [6, 7, 9, 13, 14]. This fact has been changed by anatomical variation of the right ILN in our case. We cannot found the right nerve at its usual location with dissection around the inferior artery and adjacent to ZT that a non-recurrent nerve has been identified with careful dissection above the inferior artery through Berry ligament. The parallel course of non-RLN with the artery at a cephalic position is anatomical relation of these two structures in our patient. The non-RLN has been fully isolated from its origin on the vagus nerve to laryngeal entry point. Therefore, a rare case of nerve variation has been established and documented in our operation. Nerve injury may be prevented by its full isolation based on intimate knowledge of the anatomy including all its variations which concern both the inferior laryngeal nerve and Zuckerkandl's tubercle as previously reported [15, 16]. In our patient, the non-RLN, and the ZT at slightly upper position than usual location, was a good example of combined anatomical variations. Yalçin et al. [14] have reported that only 4% of the ZT is located in the upper third of the lateral lobe. The ZT pointing the nonrecurrent nerve is the original anatomical feature of our case. The coexistence of non-RLN and bilateral ZT in the same patient, and the tubercle pointing a right non-RLN is a rare coincidence.

 The ZT and ILN have close relationship increasing their surgical importance. Careful and meticulous dissection around the tubercle, when present, is mandatory for safety of thyroid operations. Anatomical variations of ILN, especially non-RLN, are real and serious threat for operation safety. We suggest identification and full isolation of ILN before attempting excision of adjacent structures, like the Zuckerkandl's tubercle which has surgical importance for completeness of thyroidectomy.
